# CI:Mor interactions in the lysogeny switches of *Lactococcus lactis* TP901-1 and *Staphylococcus aureus* φ13 bacteriophages

**DOI:** 10.20517/mrr.2023.50

**Published:** 2024-01-19

**Authors:** Anders K. Varming, Zhiyu Huang, Ghofran M. Hamad, Kim K. Rasmussen, Hanne Ingmer, Mogens Kilstrup, Leila Lo Leggio

**Affiliations:** ^1^Department of Chemistry, University of Copenhagen, Copenhagen DK-2100, Denmark.; ^2^Department of Veterinary and Animal Sciences, University of Copenhagen, Frederiksberg DK-1870, Denmark.; ^3^Department of Biotechnology and Biomedicine, Technical University of Denmark, Kongens Lyngby DK-2800, Denmark.

**Keywords:** Lysogeny switch, temperate phage, repressor, antirepressor, corepressor, pathogen, human adaptation

## Abstract

**Aim:** To structurally characterize in detail the interactions between the phage repressor (CI) and the antirepressor (Mor) in the lysis-lysogeny switches of two Gram-positive bacteriophages, the lactococcal TP901-1 and staphylococcal φ13.

**Methods:** We use crystallographic structure determination, computational structural modeling, and analysis, as well as biochemical methods, to elucidate similarities and differences in the CI:Mor interactions for the two genetic switches.

**Results:** By comparing a newly determined and other available crystal structures for the N-terminal domain of CI (CI-NTD), we show that the CI interface involved in Mor binding undergoes structural changes upon binding in TP901-1. Most importantly, we show experimentally for the first time the direct interaction between CI and Mor for φ13, and model computationally the interaction interface. The computational modeling supports similar side chain rearrangements in TP901-1 and φ13.

**Conclusion:** This study ascertains experimentally that, like in the TP901-1 lysogeny switch, staphylococcal φ13 CI and Mor interact with each other. The structural basis of the interaction of φ13 CI and Mor was computationally modeled and is similar to the interaction demonstrated experimentally between TP901-1 CI-NTD and Mor, likely involving similar rearrangement of residue side chains during the formation of the complex. The study identifies one CI residue, Glu69, which unusually interacts primarily through its aliphatic chain with an aromatic residue on Mor after changing its conformation compared to the un-complexed structure. This and other residues at the interface are suggested for investigation in future studies.

## INTRODUCTION

Bacterial pathogens are major killers in view of rising antibiotic resistance. *Staphylococcus aureus* (*S. aureus*) is a successful commensal found in about one-third of the healthy human population, but at the same time, a dangerous opportunistic pathogen, with mortality ranging from 15%-50% in cases of *S. aureus* bacteremia^[1]^. One reason for the success of this “superbug” is the ease of acquisition of antibiotic resistance^[2,3]^. Methicillin-resistant *S. aureus* strains (MRSA) appeared within one year of first clinical use and their global spread since then is a growing challenge. MRSA strains are commonly categorized as hospital or healthcare-associated, community-associated, or livestock-associated, based on their major route of transmission. Lysogenic conversion^[4]^ and transduction^[5]^ are two of the mechanisms by which phages mediate the horizontal transfer of fitness genes between bacteria. In *S. aureus,* prophages are key contributors to pathogenesis^[6,7]^. *S. aureus* strains commonly carry between 1 and 4 prophages, and importantly, most human strains carry a prophage belonging to the Sa3int family of bacteriophages^[8]^. These phages are characterized by encoding immune evasion factors that are specific to the human immune system and thus they promote human colonization by the hosting strain^[9]^. Interestingly, livestock strains of *S. aureus* commonly do not carry Sa3int phages; however, recently, there have been observations of human infections caused by livestock strains that have acquired phages of this family^[10,11]^. The adaptation of Sa3int phages to livestock strains appears to have involved repeated integration and excision events that alter the phage attP sequence needed for site-specific integration^[12]^. Thus, Sa3int phages greatly influence the host range of MRSA strains.

Bacteriophages are found in all known biological niches and environments that can accommodate bacteria, which they outnumber by several folds^[13]^. Lytic bacteriophages kill bacteria through lysis and are promising for the treatment of antibiotic-resistant strains^[14]^. In contrast, temperate bacteriophages can enter either a lytic or a lysogenic lifecycle, where they, in addition to being lytic, can integrate their DNA into the bacterial genome as prophages, and contribute to the virulence of the host. Bi-stable genetic switches (lysis-lysogeny switches, hereon abbreviated as lysogeny switches) control whether the phage enters the lytic or lysogenic cycle^[4,15]^.

**Figure 1:**
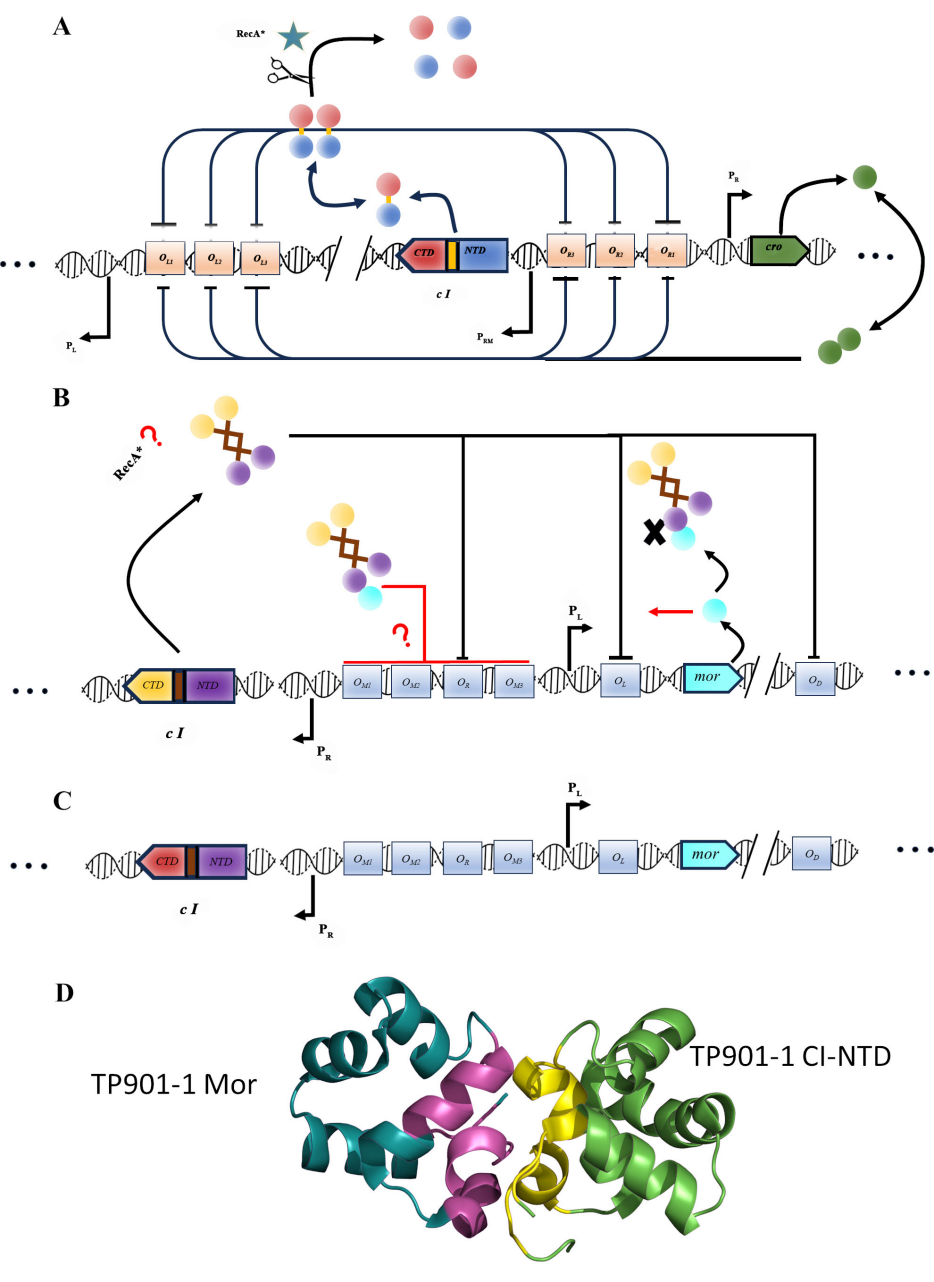
Overview of different genetic switches. (A) Simplified view of the λ phage lysogeny switch showing the interplay between CI, its antirepressor Cro, and the bacterial RecA. CI consists of two domains, the DNA-binding NTD and a protease domain at its CTD, which is activated and results in autocleavage on interaction with RecA. Here and in the diagrams below, longer bars on specific operator sites indicate higher affinity. Only CI dimers are shown, but higher oligomerization states exist and have functional roles; (B) simplified view of TP901-1 lysogeny switch. Here, Mor rather than Cro acts as a counterpart to CI. However, Mor does not seem to interact directly with DNA, but rather with the CI-NTD, blocking its binding to O_L_ and functioning as a corepressor at a composite site around O_R_. It is furthermore known that induction by the SOS response is RecA mediated, but it is not understood how, since TP901-1 CI does not undergo autocleavage, not having a protease domain or autocleavage site in its CTD. As above, higher oligomerization states of CI are not shown for simplicity; (C) overview of lysogeny switch region of φ13. The switch has a similar operator site and gene organization as TP901-1 with Mor instead of Cro; however, the C-terminal region of the CI resembles much more closely the one of λ (see color coding of domains). The φ13 switch is much less characterized at the molecular level, but it has been recently established that CI can bind to the putative operator sites with high affinity *in vitro*^[28]^; (D) overview of interaction between TP901-1 CI-NTD (in light green) and Mor (dark green) in the crystal structure (PDB 6TRI). The interface regions identified in^[24]^ are highlighted in yellow (CI) and magenta (Mor). CI: Phage repressor; NTD: N-terminal domain; CTD: C-terminal domain.

In the Gram-negative *Escherichia coli,* the best-characterized lysogeny switch is that of phage λ^[4,15]^, which is also one of the best-studied models of gene regulations. The λ switch [Figure 1A] relies on several dimers of the repressor CI (produced from the clear 1 - *cI* - gene) binding cooperatively to operator sites through their DNA-binding N-terminal domain (CI-NTD). CI binds preferentially O_L1_ O_L2_ and O_R1_ O_R2_ in the O_L_ and O_R_ operator sites, a process also involving DNA looping and long-range cooperativity, and thus blocks the lytic cycle at P_L_ and expression of the repressor Cro at P_R_. DNA damage induces the SOS response and promotes the association of the bacterial protein RecA with single-stranded DNA^[16]^. RecA filaments induce autoproteolysis of CI by its LexA-like protease C-terminal domain (CTD)^[17]^, leading to detachment from DNA, and expression of lytic genes and the repressor Cro. Cro binds then preferentially to O_R3_, blocking the expression of CI and completing the switch to the lytic cycle. Despite some similar features, for example, a general repressor DNA-binding domain structure and some analogies to the λ system, there are remarkable differences at the molecular level in lysogeny switches from different temperate phages.

Among bacteriophages of Gram-positive bacteria, one well-characterized example is the switch of TP901-1 infecting *Lactococcus lactis*, a commonly used bacterium in the dairy industry, which we have previously studied in detail at the molecular level^[18-24]^. The TP901-1 switch is encoded by a < 1 kb fragment of the phage genome [Figure 1B], including genes for the CI repressor and a Mor (modulator of repression) antirepressor (transcribed from the P_R_ and P_L_ promotors, respectively) instead of Cro. As for phage λ, CI in TP901-1 contains a DNA-binding, N-terminal domain (CI-NTD), which consists of a characteristic helix-turn-helix (HTH) structure^[19]^. Interaction between CI and DNA is at three palindromic operator sites related in sequence (O_L_, O_R_, and O_D_). Cooperative binding of the TP901-1 CI dimer to O_L_ ensures maintenance of the lysogenic, immune state, and inhibition of expression of lytic genes and *mor*. In contrast to the predominantly β-sheet fold of the λ phage CI-CTD protease domain, the TP901-1 CI-NTD is connected by a flexible linker^[22]^ to a characteristic all α-helical C-terminal domain^[20]^, within which a helical hook subdomain mediates dimerization (CTD_1_)^[23]^. TP901-1 CI does not contain a typical auto-cleavage site or a peptidase domain at its C-terminus. However, induction is still RecA dependent^[25]^ through unknown mechanisms. Furthermore, TP901-1 does not have a close functional counterpart to Cro. Instead, the lytic state depends on the Mor antirepressor. Mor also consists of a typical HTH DNA-binding domain^[24]^, in a similar way to Cro, but unlike Cro, Mor has no demonstrated high-affinity interaction with DNA on its own. Mor has a dual function in establishing the lytic state, primarily as an antirepressor of CI. Structural evidence from a CI-NTD:Mor complex^[24]^ and a CI-NTD:DNA complex^[19,23]^ strongly suggests that it functions as an antirepressor by steric hindrance, preventing binding of CI-NTD monomers in a dimer to adjacent half-sites on O_L_^[24]^. While CI is sufficient to keep the phage in the lysogenic state, direct interaction of Mor with CI is needed for the lytic state^[24]^. Evidence based on the use of switch plasmids^[21]^ and NMR titrations^[24]^ suggests that in the lytic state, the CI:Mor complex interacts with an extended composite site in the O_R_/O_M_ region, with Mor thus functioning as corepressor as well as an antirepressor for CI. A detailed analysis by NMR spectroscopy also revealed that Mor, in particular, is rather dynamic in solution and rearrangement of the structure occurs on complex formation^[24]^.

Considering the fundamental importance of lysogeny in pathogen evolution, molecular-level information is surprisingly scarce for lysogeny switches of phages infecting Gram-positive bacteria^[4]^ including pathogens. For *S. aureus*, the currently best-studied temperate phage switch is that of φ11. It resembles the switch of phage λ, but has a different arrangement of operator sites and involves an additional protein, gp07, which enhances Cro activity^[4,26]^. Furthermore, φ11 CI and Cro have only low sequence similarities with the corresponding λ proteins and significant functional differences^[27]^.

Only recently, we have characterized the lysogeny switch of φ13, a member of the Sa3int family of phages^[28]^, which, as stated above, is strongly implicated in promoting human colonization by MRSA^[29]^. The φ13 switch [Figure 1C] shares features with both phage λ and the TP901-1 switches [Figure 1B], with CI-NTD, Mor, and the operator sites being highly similar to those of the TP901-1 switch, while the CI-CTD region shows both a dimerization region reminiscent of TP901-1 and a peptidase domain and auto-cleavage site as is found in λ. We demonstrated that the genomic φ13 region corresponding to the TP901-1 switch also functions as a switch, that the lytic state is induced through the SOS response, and that φ13 CI binds to O_L_, O_R_, and O_D_^[28]^. Thus, the φ13 switch combines the auto-cleavage mechanism of lambdoid phages with a TP901-1-like dimerization domain and the Mor-mediated regulation. However, a direct interaction between CI and Mor was not demonstrated, nor were the switch components characterized structurally. In this paper, we thus focus further on the structural basis of CI:Mor interaction in the switches of TP901-1 and φ13, highlighting similarities and differences.

## METHODS

### Protein expression and purification

TP901-1 CI-NTD was expressed and purified as in^[24]^. Briefly, BL21(DE3) *E.coli* was transformed with pET30a(+) with kanamycin resistance encoding a synthetic gene for CI-NTD (residues 1-89 with a C-terminal His-tag, see construct list in Table 1). 10 mL lysogeny broth (LB) medium starter cultures were grown for 16 h, and used to inoculate 1 L of LB medium expression cultures (50 g/mL kanamycin). A final concentration of 1 mM isopropylthio-β-galactoside was added when the culture had reached an optical density (OD) of 0.5-0.6. Growth was continued overnight at 25 °C prior to harvesting the soluble fraction after sonication in lysis buffer (20 mM imidazole, 20 mM Tris pH 7.5, 1 M NaCl).

**Table 1:** Overview of crystal structures containing CI-NTD from TP901-1

**PDB code**	Sequence*	**Comment**	**Resolution**	**Space group**	**Cell**	**Ref.**
3ZHI	M**L**KTDTSNRLK QMAERNLKQV DILNLSIPFQKK FGIKLSKSTLS**A** YV**A**SVQSPDQN RIYLLAKTLGV SEAWLMG**RSH** **HHHHH**	Q45AN48A- impaired in DNA binding	1.60 Å	*P 2_1_ 2_1_ 2_1_*	a = 29.62 Å b = 43.59 Å c = 71.5 Å α = 90˚ β = 90˚ γ = 90˚	[19]
3ZHM	M**L**KTDTSNRLK QIMAERNLKQV DILNLSIPFQKK FGIKLSKSTLSQ YVNSVQSPDQN RIYLLAKTLGVS EAWLMG**RSHH** **HHHH**	Complex with DNA	2.60 Å	*P 2_1_ 2_1_ 2_1_*	a = 29.86 Å b = 64.07 Å c = 67.82 Å α = 90˚ β = 90˚ γ = 90˚	[19]
5A7L	M**Q**TDTSNRLKQ IMAERNLKQVD ILNLSIPFQKKF GIKLSKSTLSQY VNSVQSPDQNR IYLLAKTLGVSE AWLMGFDVPM VESSKIENDSEN IEETITVMKKLE EPRQKVVLDTA KIQLKEQDEQN KVKQIEDYRLS D**RSHHHHHH**	Cleaved during long crystallization period - no DNA bound	2.10 Å	*P 2_1_ 2_1_ 2*	a = 53.72 Å b = 36.01 Å c = 38.77 Å α = 90˚ β = 90˚ γ = 90˚	[20]
6TRI	MQTDTSNRLKQ IMAERNLKQVD ILNLSIPFQKKF GIKLSKSTLSQY VNSVQSPDQNR IYLLAKTLGV SEAWLMGFDVP MVESSKIENDS **HHHHHH**	Complex with Mor	2.28 Å	*P 3 2 1*	a = 94.41 Å b = 94.41 Å c = 30.56 Å α = 90˚ β = 90˚ γ = 120˚	[24]
8QAO	M**Q**TDTSNRLKQ IMAERNLKQVD ILNLSIPFQKKF GIKLSKSTLSQY VNSVQSPDQNR IYLLAKTLGV SEAWLMGFDVP MVESSKIENDS **HHHHHH**		1.29 Å	*P 4_3_ 2_1_ 2*	a = 48.4 Å b = 48.4 Å c = 87.0 Å α = 90˚ β = 90˚ γ = 90˚	This manuscript

*Non-native amino acids inserted or deviating from natural sequence are in bold. For 5A7L, the underlined amino acid is the last one visible in the crystal structure. CI-NTD: N-terminal domain of Phage repressor.

Protein was purified on an ÄKTA Purifier system (GE HealthCare/Cytiva), first by immobilizing on a 1 mL His-Trap™ HP (Cytiva) in lysis buffer, followed by a short wash in the same. Protein was eluted with 20 mM Tris, 100 mM NaCl, 500 mM Imidazole, pH 7.5. A final purification step was carried out by Size-Exclusion Chromatography on a HiLoad 26/60 Superdex 200 column with 20 mM Tris, 100 mM NaCl pH 7.5 as eluent.

Φ13 CI and φ13 Mor with N-terminal His tags (sequences in Supplementary Table 1) were produced as φ13 CI in^[28]^, following basically the same procedure as above. All synthetic genes were from Genscript and codon-optimized.

Protein concentration was determined using A_280_ measured with a NanoDrop ND-1000 UV/Vis spectrophotometer using the estimated absorption coefficient calculated from the sequence using the Expasy Protparam tool (https://web.expasy.org/protparam/). Protein was concentrated using Amicon Ultra 3kDa cutoff filters and stored frozen at -20 °C.

### Crystal structure determination of CI-NTD

Using the sitting-drop vapor diffusion technique, a JCSG+ screen (Qiagen) was set up for the TP901-1 CI NTD protein (buffer: 20 mM Tris, 100 mM NaCl pH 7.5). The Oryx 8 Protein Crystallization Robot (Douglas Instruments Ltd.) was used to set up the screen at room temperature in MRC2 Well Crystallization plates (SwissCI). The stock protein concentration used was 8.14 mg/mL. In each well, there was a reservoir volume of 100 µL (screen solution) and sitting drops of 0.3 µL The protein:reservoir ratio was 3:1 in the top drop and 1:1 in the bottom. For optimization, 2D grids for four chosen conditions were also set up using sitting drop vapor diffusion at room temperature. The crystals sent to the synchrotron were produced in the optimized condition consisting of 28% w/v PEG 4000, 0.17 M ammonium sulfate, and 15% glycerol. The protein concentration in this optimized condition was 3.3 mg/mL in 20 mM Tris pH 7.5, 100 mM NaCl (protein stock), and the protein: reservoir ratio was 3:1 in the top drop and 1:1 in the bottom, and the drop volume was 0.4 µL. Crystals were frozen in liquid nitrogen directly in mother liquor without the addition of cryoprotectant. X-ray diffraction data were collected at beamline ID23-2 ESRF, Grenoble, France. One of the datasets with 1.29 Å resolution was chosen to determine the structure. The previous TP901-1 CI-NTD structure (PDB code:3ZHI) was chosen as the reference structure to do the Molecular replacement in the CCP4 suite^[30]^ (MOLREP^[31]^). Further refinement was performed alternating computational refinement in REFMAC5^[32]^ and manual rounds in COOT^[33]^ until reaching a final R factor value of 14.97% and an R free value of 18.29%. Data statistics of X-ray diffraction and structure refinement statistics are shown in Table 2.

**Table 2:** Crystallographic statistics of the TP901-1 CI-NTD crystal structure

Beamline, date of data collection	ID23-2, ESRF, Grenoble; 21/01/2022
Auto-processed dataset used	XDSAPP
Wavelength [Å]	0.8731
Space group	*P 4_3_ 2_1_ 2*
No. of molecules/asymmetric unit	1
Cell parameters	
(a, b, c) [Å]	48.4, 48.4, 87.0
(α, β, γ) [°]	90.0, 90.0, 90.0
Resolution [Å]	43.48 - 1.29 (1.37 - 1.29)*
Completeness [%]	94.0 (69.5)
R_meas_ [%]	25.6 (375.0)
R_pim_ [%]	5.1 (198.4)
I/σ(I)	13.71 (0.48)
CC_1/2_ [%]	99.9 (21.7)
Observed reflections	891,991
Unique reflections	46,858
Redundancy	19.04
R_work_ [%]	14.97
R_free_ [%]	18.29
RMSD	
Bond lengths [Å]	0.0179
Bond Angles [°]	2.4631
Ramachandran statistics (%)^#^	
Favored	98.7
Allowed	1.3
Outlier	0

*The values in parentheses are for the highest resolution shell; ^#^Ramachandran statistics are calculated in RAMPAGE^[34]^. CI-NTD: N-terminal domain of Phage repressor.

### Modeling with AlphaFold2

AlphaFold2 (AF2) models for individual φ13 proteins were either from the Alphafold database (Q2FWP6 and Q2FWP7, model version 4)^[35]^ or constructed using the Colab version of Alphafold2^[36]^ using default parameters. TP901-1 CI-NTD was modeled using the full-length TP901-1 CI wild-type sequence with no templates. For the φ13 CI-NTD (residues 1-88)/Mor complex, the experimental complex of TP901-1 CI-NTD and Mor (PDB code 6TRI, Figure 1D) was used as template, and in parallel models were generated also without template and with automatic template recognition.

### Isoelectric focusing

Protein-protein interactions between φ13 CI and Mor were assessed using isoelectric focusing (IEF) electrophoresis under native conditions, similarly as previously for variants of TP901-1 CIΔ58 (a truncated version of CI missing the last 58 residues) and Mor^[22]^. Samples were run on a SERVALYT™ PRECOTES™ Wide Range pH 3-10 gel with SERVA IEF Marker 3-10, Liquid Mix as a protein standard. Samples were diluted to 12.7 M, and for the complex samples, CI and Mor were mixed in different ratios and incubated at 4 °C for 60 min. Samples were loaded using an applicator strip with volumes ranging from 5-15 µL in order to get comparable amounts of each protein in the different wells. The gel was run using a Multiphor II EIF chamber (Amersham Biosciences) with running conditions of 2,000 V, 12 mA, and 24 W for 180 min, and the gel was cooled to 5 °C by a thermostatic circulator. For protein detection, the gel was first fixed using 20% (w/v) trichloroacetic acid for 20 min, followed by a rinse using 3% (v/v) phosphoric acid for 5 min. The gel was stained with SERVA Violet 17 [200 mg SERVA Violet 17 mixed with 100 mL milli-Q water and 100 mL 20% (v/v) phosphoric acid] for 30 min and subsequently destained with 3% (v/v) phosphoric acid until no background staining was visible. The gel was scanned using a Gel Doc EZ system from Bio-Rad and analyzed using Image Lab software.

## RESULTS

### A new crystal structure of TP901-1 CI-NTD

As introduced earlier, the TP901-1 lysogeny switch has been characterized extensively at the structural level, and indeed, four crystal structures have already been deposited in the PDB containing the CI-NTD. Table 1 summarizes the characteristics of these structures and corresponding sequences as well as the new one presented here. However, none of the uncomplexed structures of CI-NTD corresponded to the identical sequence of the construct used for the interaction studies with Mor, which motivated a new crystallization effort. The new X-ray structure, which has by far the highest resolution for a TP901-1 CI-NTD structure obtained so far, resembles the overall previous structures of TP901-1 CI-NTD with the typical HTH motif. Generally, the structure is of high quality. It was determined at a maximum resolution of 1.29 Å with a final R factor of 14.97% and R free of 18.29%. 98.7% of residues are in the favored regions of the Ramachandran plot, no residues lie in outlier regions, and stereochemical parameters are good. However, several exposed side chains have alternate conformations (Met1, Ser6, Gln11, Ile12, Met13, Glu15, Ile23, Ser41, Ser44, Ile58, Glu69, Met73, Met79, and Val80) or are not fully visible in the electron density maps. The termini, in particular, were extremely difficult to place. The C-terminus beyond residue 80 is particularly problematic, as could be expected in view of previous results indicating the start of the linker to the CTD in this region. However, there was definite disordered electron density beyond this point, which, after many attempts, was modeled as res 81 connected to the rest of the chain and two additional residues (83-84) after a one-residue gap. As modeled, the positions of res 81 and the res 83-84 segment are not compatible with each other, but each represents one of presumably multiple conformations assumed by the chain in this area. Atomic details in this region cannot be relied upon and some electron density remains unmodeled.

### Comparison of TP901-1 CI-NTD crystal structures in the interface area

We have previously analyzed in detail the dynamics of TP901-1 Mor based on NMR data and the CI-NTD:Mor interface as revealed by the crystal structure, and discovered that rearrangement and dynamics of aromatic residues at the Mor interface with CI-NTD are important for the formation of the complex^[24]^. The N-terminus of Mor is involved in the interface with CI, including aromatic residues Tyr3 and Tyr5. Furthermore, in the NMR structure of Mor alone, the conformation of Trp43 is incompatible with the crystal complex with CI-NTD. Thus, molecular recognition dynamics involving repacking of two aromatic rings (Tyr5 and Trp43) seems to be required for interaction with CI-NTD.

**Figure 2:**
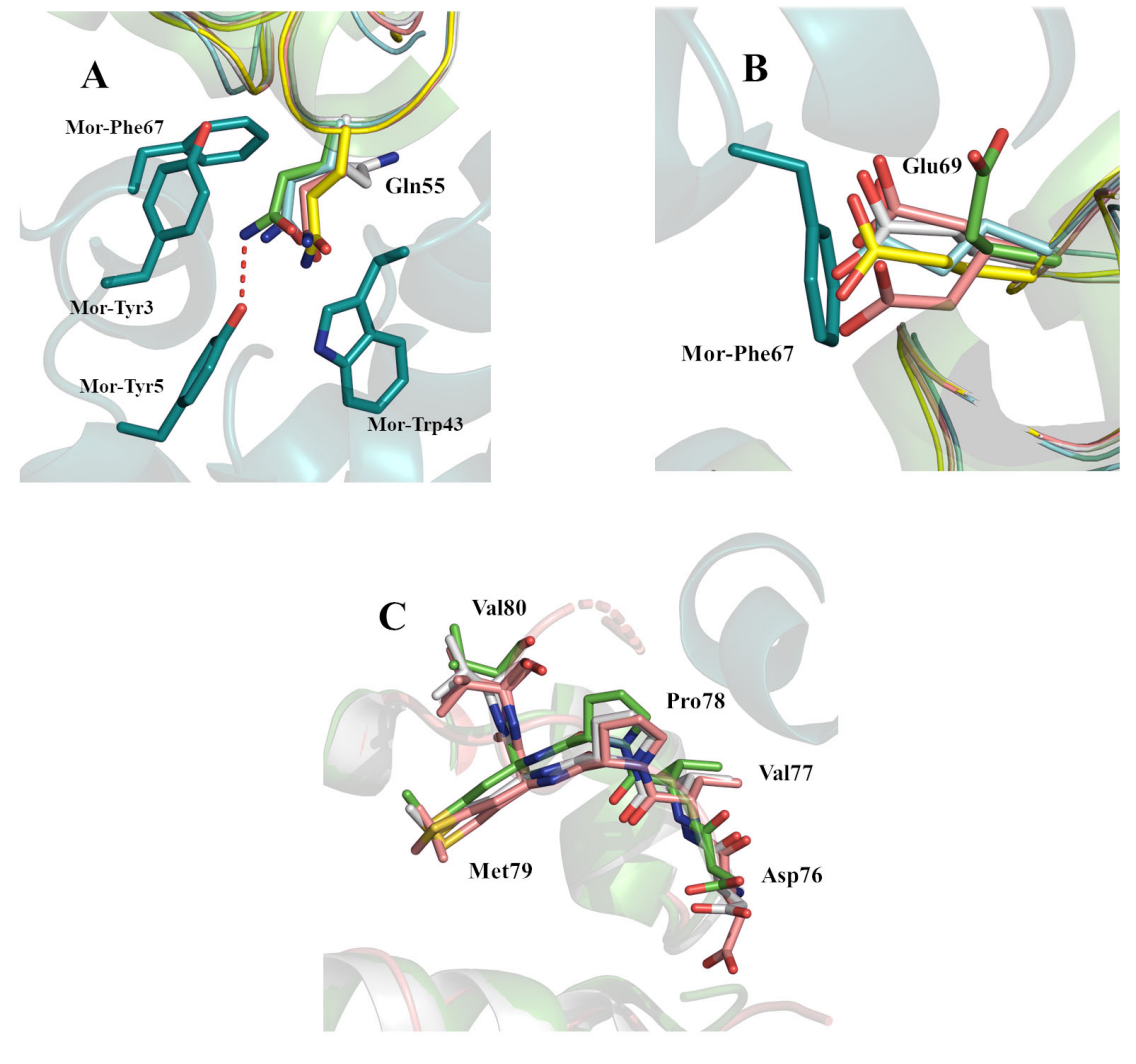
Comparison of different TP901-1 CI-NTD structures. (A) Aromatic residues Tyr3, Tyr5, Trp43, and Phe67 of TP901-1 Mor form a cavity in which Gln55 from CI must fit for complex formation; (B) the conformation of Glu69 in the structures of CI without Mor would cause severe clashes with Phe67 of Mor in the complex; (C) the structures of the C-terminal region from Asp76 to Val80 are similar in CI-NTD alone (5A7L, 8QAO from this manuscript) and in the complex with Mor (6TRI). The CI-NTD:Mor complex structure is shown in light green (CI) and teal (Mor) cartoon representation with green sticks for highlighted side chains. The shown sticks are from CI-NTD containing structures 3ZHI (light blue), 3ZHM (yellow), 5A7L (grey), and 8QAO from this manuscript (salmon). CI-NTD: N-terminal domain of Phage repressor.

Here, we focus on the reorientation of residues that might occur in the TP901-1 CI-NTD when interacting with Mor, based on a comparison of the various crystal structures in Table 1. Interface residues have previously been identified in the N-terminal region (2-**Q**TDT-5), C-terminal region (68-SEAWLMGFDVP-78), and the first half of α-helix 4 (52-SPDQNRIY-59)^[24]^. The N-terminus, which differs considerably in different structures (also as the native Q marked in bold above is sometimes a K due to cloning artifacts), has only a minor involvement in complex formation, so it will not be discussed in detail. Gln55, Glu69, and Met73 (underlined in the sequences above) stand out as interface residues taking on different conformations in different crystal structures. CI Gln55 [Figure 2A] in the complex has to fit snugly into a cavity formed by aromatic residues Tyr3, Tyr5, Trp43, and Phe67 of Mor, where it comes into hydrogen bonding distance of Tyr5. Gln55, which is overall well defined in the electron densities of the respective structures, needs to generally make a minor rearrangement to fit in the complex, compared to the larger rearrangements seen by some of the aromatic side chains forming the pocket in Mor. The position of CI Glu69 in all crystal structures except for the CI:Mor complex [Figure 2B] is always pointing in the same direction and is reasonably well-defined in the respective electron densities, which could be due to the residue being stabilized in this position through interaction with side/main chain amines of crystal neighbors. In the most recent high-resolution structure, there are no such interactions and two conformations were necessary for adequate modeling of the side chain. An omit map where Glu69 was modeled as Ala [Supplementary Figure 1] clearly indicates that the Glu69 side chain density would clash with Phe67 of Mor in the complex; thus, a considerable rearrangement is required on complex formation. In the Mor NMR structure ensemble (PDB code 6TOE), the Phe67 side chain can be in alternate conformations, all clashing with Glu69 in the uncomplexed CI-NTD crystal structures.

The extreme C-termini of the CI-NTDs are difficult to compare as the sequences differ here due to the presence of tags and different construct lengths. However, we note that the C-terminal region Asp76-Val80 is very similar in the CI:Mor complex and in the only two other structures that contain this region [Figure 2C]. In the most recent structure, the C-terminus folds against the rest of the protein beyond Val80 and becomes difficult to trace, but this is unlikely to be the case in the full-length protein, as this region is the linker to the dimerization region.

To satisfy our curiosity, we produced a model of full-length TP901-1 CI by AF2 without using a template structure and compared the CI-NTD region to the CI-NTD region in the experimental complex. Interestingly, in the computational model, both Gln55 and Glu69 side chain positions are compatible with complex formation with Mor, though clashes arise at the N-terminus of CI.

### Purification of φ13 Mor

**Figure 3:**
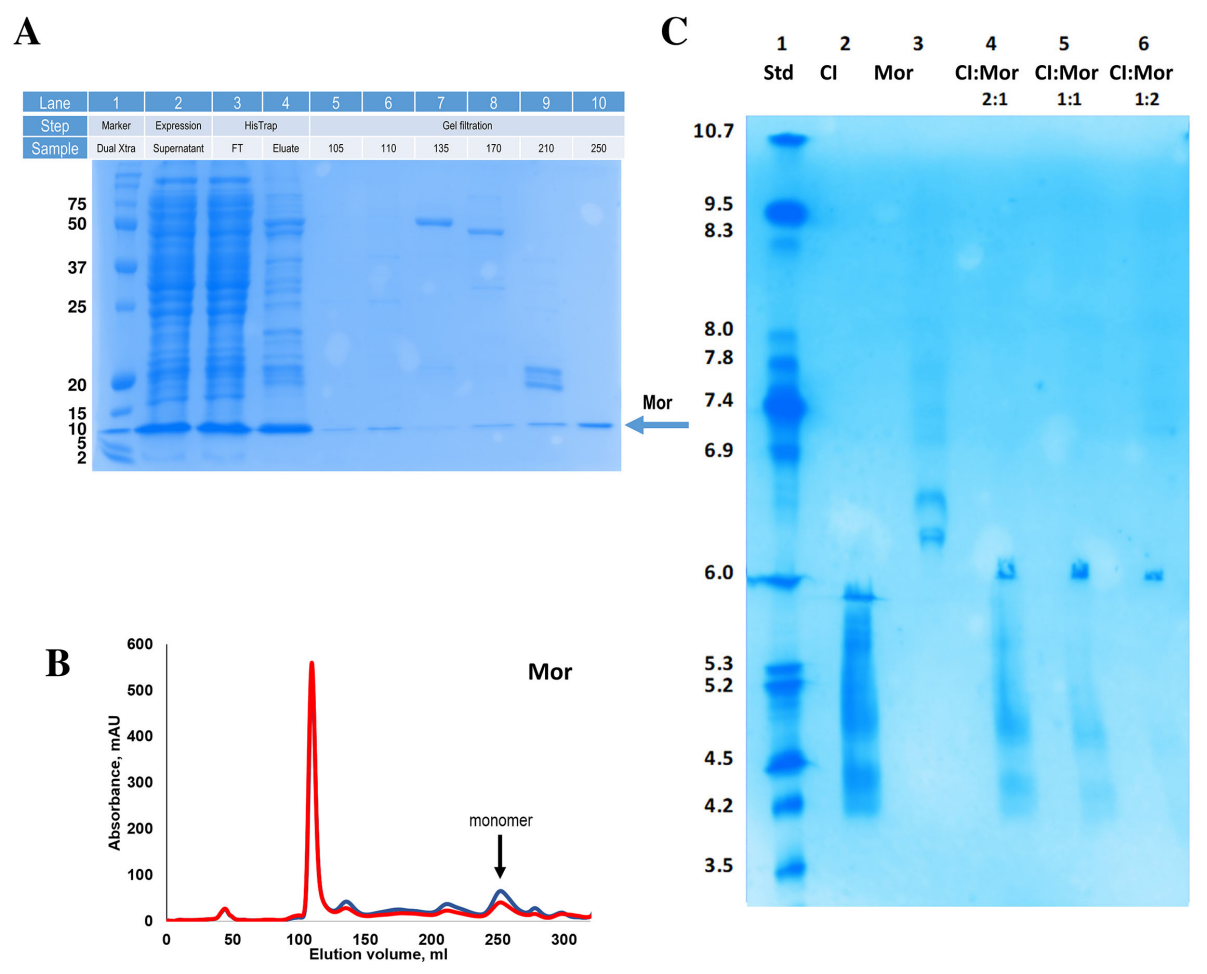
Purification of φ13 Mor and characterization of interaction with φ13 CI. (A) Analysis of Mor purification progress on 15% SDS-PAGE, showing a single band for Mor in SEC elution fractions. Each type of fraction loaded is indicated above the lane. Fractions named FT contain the flowthrough from HisTrap affinity chromatography, while numbers listed below gel filtration step indicate the elution volume of each fraction; (B) SEC chromatograms of φ13 Mor. The absorbance at 280 and 260 nm are shown in blue and red, respectively, and the estimated oligomeric state is marked by an arrow; (C) IEF electrophoresis of CI and Mor alone and in complex. The pH standard values are listed on the left and the protein sample composition for each lane is above. CI: Phage repressor; SEC: selected gel filtration; IEF: isoelectric focusing.

We have previously purified and partly characterized φ13 CI^[28]^. Here, we produce, purify, and characterize for the first time φ13 Mor [Figure 3]. As observed for TP901-1 Mor, φ13 Mor elutes as a monomer in gel filtration [Figure 3B] based on a comparison with a standard calibration curve.

### Interaction of φ13 CI and Mor in solution

The possible interaction between φ13 CI and Mor was studied using isoelectric focusing (IEF) electrophoresis. CI and Mor have theoretical pI values of 5.24 and 8.63, respectively, and a complex between CI and Mor would thus be expected to have an intermediate pI. Each protein was run alone (lanes 2 and 3 in Figure 3C) and in complex with its counterpart at different ratios (lanes 4-6 in Figure 3C). Both protein bands are smeared, making it difficult to establish a specific experimental pI. CI shows a stronger band just below the standard at pH 6.0, while Mor shows at least two strong bands between the standards at pH 6.0 and pH 6.9. This could indicate conformational heterogeneity, consistent with a highly dynamic molecule as previously observed for TP901-1 Mor. We do, however, see a clear difference between CI and Mor alone and in complex. The bands arising from Mor in lane 3 disappear when mixed with CI in a 2:1 CI:Mor complex, indicating that Mor interacts with CI. However, this complex also indicates an excess of CI as the bands arising from CI in lane 2 are still visible, albeit less intense. This excess decreases in the 1:1 ratio complex and seems to disappear completely in the 1:2 CI:Mor complex, suggesting that most CI and Mor in solution have formed a complex, and it is a well-defined complex, since a single band is observed. The binding stoichiometry of the CI:Mor interaction cannot be concluded unambiguously from these results, also due to the uncertainties in the estimation of protein concentration, but it is clear that φ13 CI and Mor interact in solution, supporting the notion that CI and Mor in φ13 regulate the genetic switch in a similar way to their homologs in TP901-1.

### Computational modeling of the φ13 CI-NTD:Mor complex

After demonstrating through experiments that there is interaction between φ13 CI and Mor, our focus shifted to modeling the complex. Given the high sequence conservation between CI-NTD and Mor in TP901-1 and φ13 (> 60% sequence identity), the interactions may be expected to be strikingly similar. Thus, we initially constructed a model of the complex, by superposing models of φ13 CI and Mor from the AF Database to the experimental structure of the TP901-1 complex. However, this resulted in severe clashes at the interface between φ13 CI-NTD and Mor [Supplementary Figure 2], a major one being CI Tyr75 (corresponding to Phe75 of TP901-1) clashing with several residues of Mor including Trp49 (TP901-1 Leu49). The overall Molprobity clash score calculated at the Swissmodel server (https://swissmodel.expasy.org/assess) was 27, with most clashing residues situated at the interface. Given that the TP901-1 CI:Mor interaction involves several termini and side chain rearrangements, these clashes are perhaps not surprising, but they diminish confidence in the results.

Previous attempts to generate a heterodimer model using an earlier version of AF2 yielded a very different interaction interface and heterodimers compared to the experimental CI-NTD:Mor heterodimer for TP901-1. Given the continuous progress in the AF2 computational pipelines, we recently attempted again, both using 6TRI as a template and in no template mode, in both cases resulting in complexes similar to the experimental TP901-1 heterodimer. The clash scores of unrelaxed models were still high (22-24), but now the clashes were not concentrated at the interface. Relaxation of the best-scoring AF2 model produced with no template yielded a highly favorable clash score (1.83) and resolved the interface problems, leading to the creation of a credible model for the CI-NTD:Mor interface in φ13, which is presented here for further discussion and is available as supplementary material.

### Analysis of the φ13 CI-NTD:Mor interface

**Figure 4:**
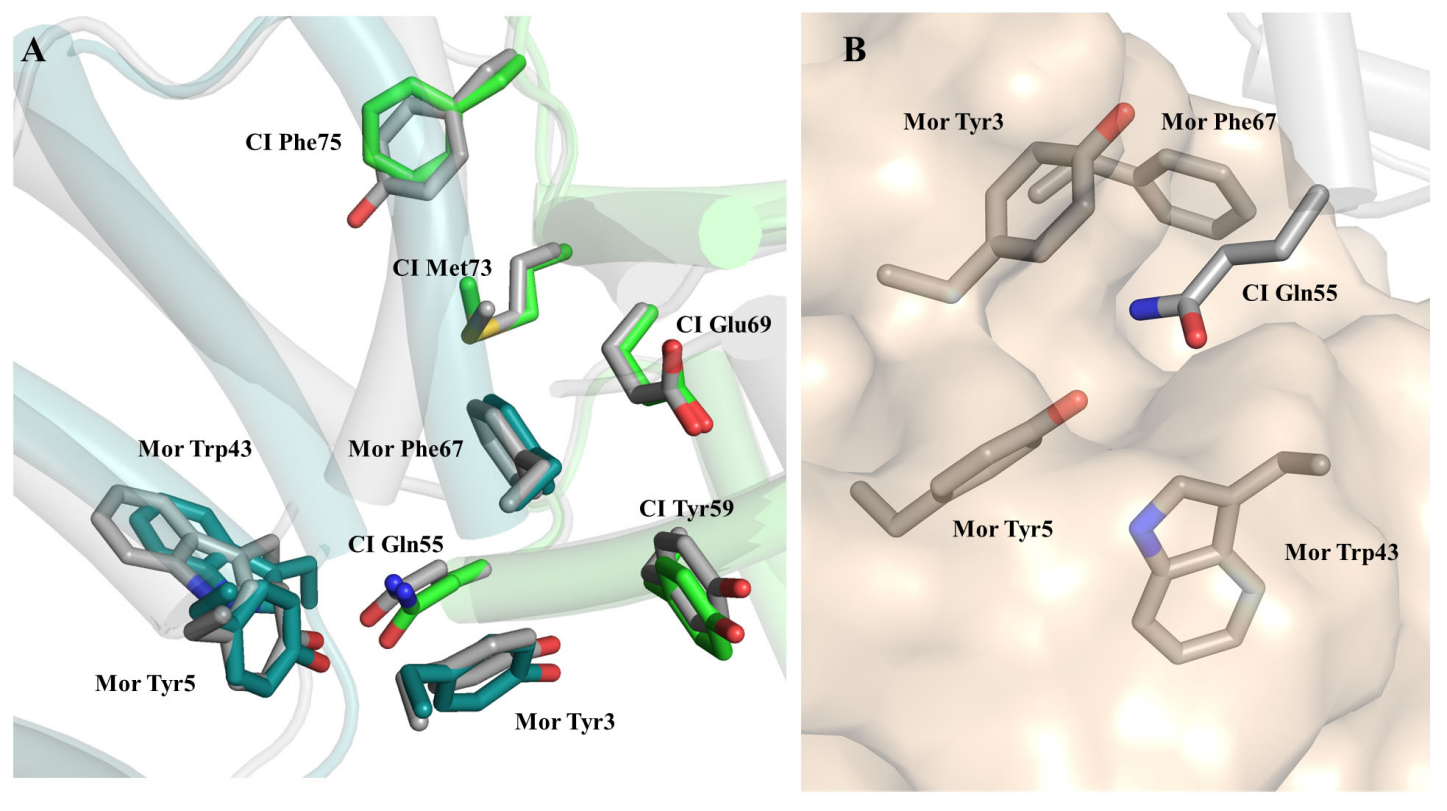
Computational model of the φ13 CI-NTD:Mor complex. (A) Superposition of the TP901-1 experimental CI-NTD:Mor complex (PDB code 6TRI, color scheme as in previous figures) with the φ13 CI-NTD:Mor complex obtained with AF2 (in grey). The extreme N- and C-terminus of Mor are omitted for clarity. Helices are shown as cylinders. Side chains of interface residues mentioned in the text are shown as sticks representation. Label numbering is for TP901-1 proteins; (B) Close-up of the φ13 CI-NTD:Mor complex highlighting the Mor pocket in which Gln55 from CI fits. A semitransparent surface is shown for Mor. CI-NTD: N-terminal domain of Phage repressor.

A close-up of the interface region is shown in Figure 4. Several residues at the interface (Gln55, Tyr59, Met73, and Glu69) are conserved in CI from the two species [Figure 4A]. Residue 75 is Phe in TP901-1 and Tyr in φ13. In TP901-1^[24]^, the substitution of Met73 to Ala had a great impact *in vitro* and *in vivo*, while the substitution of Phe75 to Ala had less impact, and the substitution with Tyr only a mild impact, which is compatible with the residue being a Tyr in the φ13 system. Substitution of Gln55 had a large effect *in vivo*, whereas substitutions of Tyr59 and the previously discussed Glu69 have not been investigated, but they undoubtedly warrant further investigation. Tyr3, Tyr5, Trp43, and Phe67 are conserved interface aromatic residues on Mor, forming a pocket in which Gln55 from CI can fit [Figures 2A and 4B] in both phages. Substitution of Tyr5 and Phe67 had large effects in TP901-1 *in vivo*, while the role of Tyr3 and Trp43 was not investigated by mutagenesis. The model for the complex suggests several further studies of the φ13 switch, for example, using the switch plasmids in^[28]^, to understand the importance of these residues.

## DISCUSSION

The lactococcal TP901-1 lysogeny switch is one of the best studied at the molecular level from Gram-positive bacteria phages. As stated above, the mechanism for maintaining the lysogenic state is well understood and depends on the dimeric form of the CI repressor binding to its operator sites with high affinity^[21,24]^. The Mor protein has the DNA binding HTH motif but is unable to bind to DNA by itself; however, through complex formation with Mor in the lytic life cycle, CI becomes unable to bind to both of the half-sites in O_L_ due to steric hindrance. Furthermore, the CI:Mor complex appears to be able to bind to a composite binding site, thereby repressing the lysogenic promoter^[21,24]^.

Because of its dual involvement in the Mor function as antirepressor and corepressor, the CI:Mor complex is central to the TP901-1-like phage switches. Previously, the kinetics of CI:Mor complex formation was analyzed by isothermal titration calorimetry, showing that the dissociation constant was reasonably high, around 650 nM^[24]^. Mutant switches with altered amino acids in the CI:Mor interface were constructed, based on the crystal structure of the CI-NTD:Mor complex. Using switching frequencies as an assay for CI:Mor complex formation, the CI:Mor interface mutants showed both increased and reduced complex formation. Though the effect was smaller than for other residues, increased complex formation (low lytic switch frequency) was seen for CI-Phe75Ala and CI-Phe75Val substitutions, while decreased complex formation was seen for CI-Phe75Ile and CI-Phe75Tyr substitutions (increased lytic switch frequency). As in φ13, the corresponding residue to Phe75 is a Tyr; this could indicate that in φ13, the CI:MOR complex is slightly weaker; however, the interacting residues on Mor are not conserved between TP901-1 and φ13. This may have functional implications that are not yet understood, though as we demonstrate here, the φ13 CI:MOR complex can definitely form *in vitro*.

The refined crystal structure of the CI-NTD in the present study enabled us to see the changes in CI conformation upon CI:Mor complex formation in greater detail. No large changes in the location of Phe75 were observed between the CI-NTD and CI-NTD:Mor structures, and neither in the following amino acids Asp76 to Val80. Instead, the most notable rearrangement upon complex formation is Glu69, which forms an unusual interaction with the aromatic ring of Phe67 in Mor through its aliphatic chain. In the solution NMR structure ensemble of Mor alone, Phe67 assumes several conformations, about half very similar to the one in the crystal complex of CI-NTD and Mor, but all clashing with CI-NTD Glu69 in its uncomplexed form. The functional role of Glu69 residue was not previously investigated and would be an interesting topic for future mutagenesis studies.

Recently, the lysogeny switch of the *Sa* phage φ13 - important for human colonization by *S. aureus* - has been partly characterized^[28]^. This switch contains elements similar to both the TP901-1 and λ lysogeny switches. In particular, the sequence similarity of CI-NTD and Mor to the TP901-1 counterparts suggests similar interaction mechanisms. It has therefore been important to establish that φ13 CI and Mor can also form stable complexes, which we demonstrate here. Furthermore, through the appropriate use of AF2 modeling, we have been able to model and analyze the φ13 CI-NTD:Mor interface and suggest further areas of investigation.

Recent work has shown that in addition to the interplay between CI, Mor, and RecA, host-specific factors and additional phage regulatory genes are also involved in determining the mobility of φ13 and other *S. aureus* phages^[37]^. This opens new possibilities for discovering important regulatory mechanisms beyond the CI:Mor switch. However, it is important to remember that even the basic switching mechanism and its interplay with host factors is not yet understood in φ13, and there is a danger in assuming similarity to other systems. The structural conservation certainly suggests strong functional conservation in the switch mechanisms of TP901-1 and φ13. Thus, we could expect a similar mechanism for the antirepressor function of MOR in φ13 as in TP901-1, implying that Mor binding to CI interferes with high affinity binding to O_L_. However, it is puzzling then that the φ13 switch should also require a λ-like CI protease domain for its function, since the TP901-1 switch does not. Therefore, future work needs to ascertain experimentally if either or both mechanisms (CI:Mor binding and/or CI autocleavage) are at play during the establishment of the lytic state in φ13. Furthermore, the presumed additional function of CI and Mor as corepressors of P_R_ is still poorly characterized. In TP901-1, Mor Arg31 has been implicated in the binding of the CI:Mor complex to the composite operator site, such that its mutation to Ala resulted in 100% lysogeny. This residue is structurally conserved in φ13, where it might have a similar role. However, the way in which the complexes interact with the operator sites to repress transcription of the lysogenic genes is still a significant question mark for both phages.

For φ13, determining the details of the switch will enable an understanding of which conditions promote the excision of the phage and which cells it may successfully integrate into. As φ13 and Sa3int phages promote human colonization, their establishment in *S. aureus* strains such as those of animal origin can cause host jumps of strains, as has recently been observed for livestock strains infecting humans^[10]^. Knowledge of the molecular details of φ13 lysogeny and the mechanistic details behind phage excision may be leveraged in the future to limit dissemination of the phage, thereby reducing the human risk of *S. aureus* infections. Furthermore, similar lysogeny switches as in φ13 have been detected in several streptococcal and enterococcal species^[21]^, where prophages are also increasingly linked to pathogenesis^[38,39]^; thus, a molecular understanding of such Mor-containing switches has even wider implications.

## DECLARATIONS

### Availability of data and materials

The TP901-1 and CI-NTD crystal structure and related data were deposited at the Protein Data Bank with code 8QAO. The computational model for φ13 CI-NTD:Mor complex is available in Supplementary Materials.

### Copyright

© The Author(s) 2024.
