# Supplementary material for: CI:Mor interactions in the lysogeny switches of Lactococcus lactis TP901-1 and Staphylococcus aureus φ13 bacteriophages
Source: Microbiome Res Rep. 2024 Jan 19;3(2):15. doi: 10.20517/mrr.2023.50 (PMC11149083; doi:10.20517/mrr.2023.50)
Supplement: Supplementary file 1 [file mrr-3-2-15-SupplementaryMaterials.zip › mrr-3-2-15-SupplementaryMaterials/Supplementary Materials.pdf]

Supplementary Materials

CI:Mor interactions in the lysogeny switches of *Lactococcus lactis* TP901-1 and *Staphylococcus aureus* φ13 bacteriophages

Anders K. Varming[1], Zhiyu Huang[1], Ghofran M. Hamad[1], Kim K. Rasmussen[1], Hanne Ingmer[2], Mogens Kilstrup[3], Leila Lo Leggio[1]

[1]Department of Chemistry, University of Copenhagen, Copenhagen DK-2100, Denmark.
[2]Department of Veterinary and Animal Sciences, University of Copenhagen, Frederiksberg 1870, Denmark.
[3]Department of Biotechnology and Biomedicine, Technical University of Denmark, Kongens Lyngby 2800, Denmark.

Correspondence to: Prof. Leila Lo Leggio, Department of Chemistry, University of Copenhagen, Universitetsparken 5, Copenhagen DK-2100, Denmark. E-mail: leila@chem.ku.dk

Supplementary Table 1. Sequences of the φ13 constructs used. His-tag sequences are highlighted in red; TEV protease cleavage sequences are highlighted in green and mutated residues are highlighted in cyan.

**CI-WT with TEV cleavage site and His-tag**
MKHHHHHHPMSDYDIPTTENLYFQGMREKVSNRLKHIMKIRNLKQVDIINKSKPYQKKLGISLSK<br>STLSQYINDVQSPDQDRIYLLSKTLNVGEAWLMGYDVDSYRVPDEERQDETIMSKINNIFSQLTP<br>PRQENVLNYANEQLEEQNKVTSIDGYKESKLVSYIACGATGAGIGEELYDDILHEEVFFKEDETP<br>SNADFCILVNGDSMEPMLKQGTYAFIKKEDSIKDGTIALVVLDGVSLIKRVDICEDYINLVSLNP<br>KYDDIKVASFSDIKVMGKVVL
**Mor with TEV cleavage site and His-tag**
MKHHHHHHPMSDYDIPTTENLYFQGMCYDYSRLSGKIVEKYGTQYNFAIAMKLSERSLSLKLNGK<br>VGWKDSEIWKAIQLLDIPVEKIHLYFFKEKVHVI

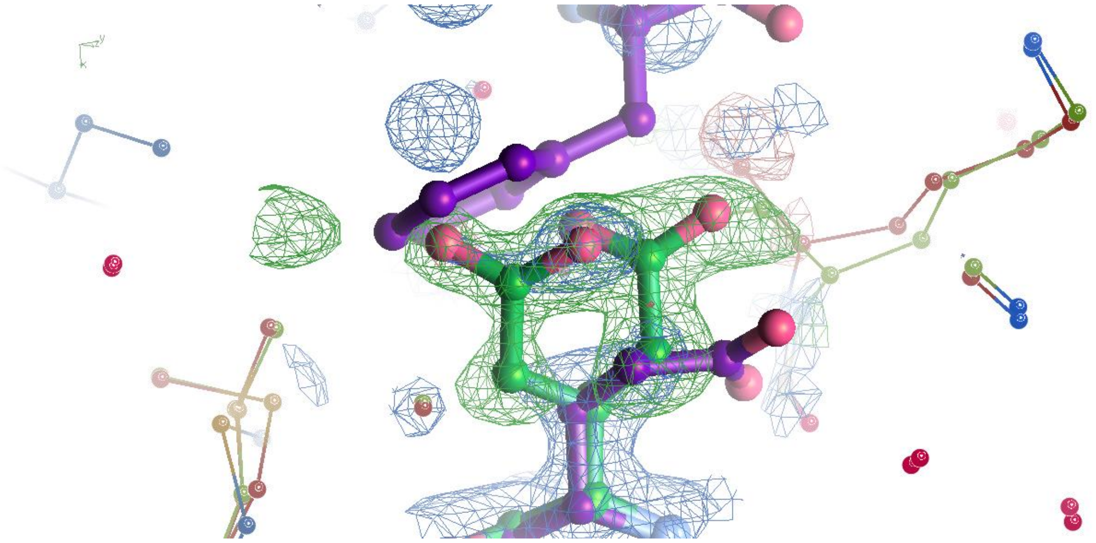

Supplementary Figure 1. Omit map for Glu69 in the current TP901-1 CI-NTD structure (green) after modeling as Ala. The 2 Fobs-Fcalc electron density map is shown at 1.5σ in blue, while the positive difference electron density is shown in green at 3σ. The structure is superposed on the TP901-1 CI-NTD:Mor complex (PDB 6tri), showing CI-NTD Glu69 and Mor Phe67 in purple.

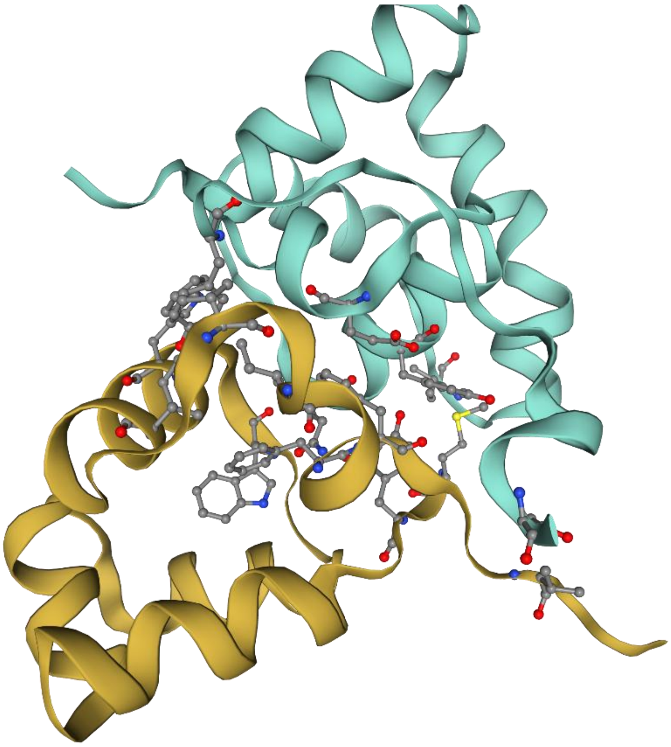

Supplementary Figure 2. Interface clashes in the initial φ13 CI-NTD:Mor computational model constructed by superposing AF2 models of the individual φ13 proteins onto the experimental TP901-1 complex (PDB code 6TRI). Clashes were detected and visualized at the https://swissmodel.expasy.org/assess.

Supplementary Model. Coordinates of the best final model of φ13 CI-NTD:Mor are available as supplementary material.
